# Phages Shape the Transformation of Organic Matter During Composting

**DOI:** 10.1111/1751-7915.70291

**Published:** 2025-12-21

**Authors:** Yuanyuan Bao, Jan Dolfing, Ruirui Chen, Chongwen Qiu, Jianwei Zhang, Xin Zhou, Liang Liu, Yiming Wang, Xiangui Lin, Youzhi Feng

**Affiliations:** ^1^ State Key Laboratory for Development and Utilization of Forest Food Resources Nanjing Forestry University Nanjing China; ^2^ Co‐Innovation Center for Efficient Processing and Utilization of Forest Resources, College of Chemical Engineering Nanjing Forestry University Nanjing China; ^3^ Faculty of Engineering and Environment Northumbria University Newcastle upon Tyne UK; ^4^ Haina Research Institute of Guangdong Haina Agricultural co., Ltd. Huizhou China; ^5^ Institute of Agricultural Resources and Environment, Jiangsu Academy of Agricultural Sciences Nanjing China; ^6^ State Key Laboratory of Soil and Sustainable Agriculture, Institute of Soil Science, Chinese Academy of Sciences Nanjing China; ^7^ Jiangsu Collaborative Innovation Center for Solid Organic Waste Resource Utilization Nanjing China

**Keywords:** bulk metagenomes, life‐history strategies, manure composting, phage ecology, viral shunt

## Abstract

Microorganisms drive the biotransformation of dissolved organic matter (DOM) during organic wastes composting, yet the role of phages with different lifestyles (i.e., temperate and virulent) in this process remains poorly understood. Here, bulk metagenomic sequencing combined with electrospray ionisation (ESI) Fourier transform ion cyclotron resonance mass spectrometry (FT‐ICR MS) was used to investigate the dynamics of temperate and virulent phage communities, microbial functional traits represented by the growth yield (Y)–resource acquisition (A)–stress tolerance (S) life‐history strategies (Y‐A‐S) framework, and molecular changes in DOM composition, as well as their potential linkages during the composting of a rice chaff and chicken manure mixture. Our results revealed that the ratio of temperate/virulent phage, microbial Y/A strategy, and microbial‐/plant‐derived DOM components exhibited highly consistent dynamic patterns, all peaking during mid‐composting stage when temperatures are elevated and remaining low at the initial and final stages. Random forest analysis further identified the ratio of temperate/virulent phages and the microbial Y/A strategy as key predictors of the variance in microbial Y/A trade‐offs and microbial−/plant‐derived DOM components, accounting for 10% and 13% of the explained variance, respectively. Together, our results demonstrate that an increased prevalence of temperate phages promoted the microbial Y‐strategy and the accumulation of microbial‐derived DOM components, while a greater dominance of virulent phages favoured the A‐strategy and plant‐derived DOM enrichment. These findings offer new insights into the ecological role of phages in mediating material transformation during organic waste composting.

## Graphical abstract

This study demonstrates that previously overlooked phages drive microbial life‐history trade‐offs, shape organic matter transformation, and provide implications for soil carbon stability in agricultural ecosystems.

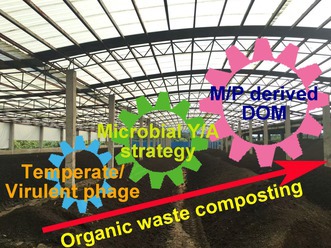

## 1 Introduction

Composting is an effective biotechnological approach to convert organic wastes, such as crop residues and animal manure, into valuable organic fertilisers, promoting soil organic carbon sequestration and crop growth (Liang et al. 2017; Zheng et al. 2021; Xu et al. 2024; Tu et al. 2025). Microbial communities are the main drivers of organic matter (OM) transformation in this process. Their growth and metabolic activity directly influences the quality of the final compost product (Bao et al. 2021; Wang et al. 2024). Thus far, research has primarily focused on the bacteria and fungi involved (Li et al. 2025; Liu et al. 2025; Tu et al. 2025). Viruses, their ecological functions, and the mechanisms underlying their effects have been largely overlooked (Zhou et al. 2025; Zhu et al. 2025).

The transformation of OM is associated with two microbial pathways (Liang et al. 2017, 2019): the in vivo pathway, in which microorganisms assimilate labile carbon released during OM decomposition into their biomass, which leads to the formation of microbial residues after cell death (Liang et al. 2017); and the ex vivo (extracellular) pathway, where microorganisms secrete enzymes to degrade OM (e.g., crop residues), which promotes the formation of plant‐derived components (Schmidt et al. 2011). These two pathways are primarily associated with the Y‐ and A‐strategies within the microbial Y‐A‐S life‐history strategies framework: the in vivo pathway corresponds to high‐yield (Y) strategies, whereas the ex vivo pathway aligns with resource‐acquisition (A) strategies (Malik et al. 2020). In contrast, stress‐tolerance (S) strategies are not directly linked to either pathway but represent an independent axis characterised by traits that enhance survival under environmental stress (e.g., extreme temperature, pH, salinity, or drought). The trade‐offs between microbial Y‐A‐S life‐history strategies are shaped by external environmental conditions, such as nutrient availability and environmental stress (Frank 2010; Sinsabaugh et al. 2013; Manzoni et al. 2014). Factors such as sustained warming, drought, or nutrient limitation have been shown to instigate microbes to reallocate resources among individual growth (Y‐strategy) and resource acquisition (A‐strategy) (Li et al. 2022; Yang et al. 2024). Consequently, shifts in environmental conditions can regulate the trade‐offs between microbial Y‐A‐S life‐history strategies, altering energy allocation between the in vivo and ex vivo pathways, ultimately influencing the balance between microbial‐ and plant‐derived components during OM transformation and affecting compost quality in a given environment. However, this hypothesis has not yet been examined. Moreover, current studies of Y‐A‐S life‐history strategies are focused on abiotic influences. The role of biotic factors—such as phages—remains largely unexplored.

It is known that nearly all cells on Earth are associated with viruses, with phages being the dominant viral type in the environment (Suttle 2007; Helsley et al. 2014; Jansson and Wu 2023). The lytic and lysogenic cycles of phages can regulate host growth (linked to the Y‐strategy) (Albright et al. 2022) and shape host‐associated ecological functions (linked to the A‐strategy) (Quesada et al. 2012; Huang et al. 2021; Zhou et al. 2023). These effects are primarily mediated through two types of virus‐host interactions: (1) the “kill‐the‐winner” model mediated by virulent phages (Winter et al. 2010), and (2) the “piggyback‐the‐winner” model mediated by temperate phages (Knowles et al. 2016; Silveira and Rohwer 2016). In the former case, virulent phages lyse dominant host cells, thereby inhibiting the community‐level Y‐strategy characterised by rapid microbial growth (Braga et al. 2020). In contrast, temperate phages can integrate into host genomes through lysogeny and express auxiliary metabolic genes (AMGs) that enhance host metabolic capabilities and facilitate the A‐strategy, associated with efficient resource acquisition and utilisation (Wu, Davison, et al. 2021). For instance, some temperate phages carry AMGs involved in lignocellulose degradation, significantly enhancing the lignocellulose‐degrading capabilities of their microbial hosts (Emerson et al. 2018). Therefore, phages may regulate the ecological trade‐offs of microbial life‐history strategies through the “kill‐the‐winner” and “piggyback‐the‐winner” mechanisms, thereby influencing the prevalence of microbial‐ and plant‐derived components during composting. Ultimately, phages may play an advantageous role in shaping the quality of the final compost product. To the best of our knowledge, however, this expectation has not yet been tested. Based on the above insights, we hypothesize that: (1) increased presence of virulent phage suppresses the community‐level Y‐strategy, thereby reducing microbial‐derived components in the final compost product; and (2) increased presence of temperate phage promotes the A‐strategy, enhancing plant‐derived components.

To test these hypotheses, we utilised a previously established organic waste composting system fed rice chaff and chicken manure (Bao et al. 2021). Bulk metagenomic sequencing was conducted to analyse and characterise the dynamics of phage community composition and microbial functional attributes throughout the composting process. Since composting is closely linked to microbial activities and most materials pass through the aqueous phase, dissolved organic matter (DOM) is considered as the bioavailable and active organic fraction and is widely used to investigate OM transformation (Yu et al. 2019). Electrospray ionisation (ESI) Fourier transform ion cyclotron resonance mass spectrometry (FT‐ICR MS) was applied to analyse molecular‐level changes in DOM composition during composting. Among the detected compounds, aliphatic molecules were classified as microbial‐derived, whereas terrestrial organic materials—such as lignins and tannins—were classified as plant‐derived (Stubbins et al. 2010; Antony et al. 2014; Feng et al. 2016). We aimed to (1) reveal the temporal dynamics of temperate and virulent phage abundances, microbial Y‐A‐S life‐history strategies trade‐offs, and microbial‐ and plant‐derived DOM components during composting; and (2) assess the role of phage‐driven shifts in Y‐A‐S life‐history strategies in regulating DOM transformation. This study will advance our understanding of microbial mediated OM transformation during organic waste composting, particularly as mediated by phages, and offer insights for developing phage‐based strategies to improve compost quality.

## 2 Materials and Methods

### 2.1 Composting Set‐Up, Chemical Analysis and Bulk Genomic DNA Extraction of Composting Samples

The composting set‐up, chemical analysis and DNA extraction of composting samples have been described in detail in a previous study (Bao et al. 2021). In brief, the composting experiment was conducted using a mixture of rice chaff and chicken manure, adjusted to a C/N ratio of 25:1 and ~60% moisture. Composting was carried out for 45 days with mechanical turning and temperature monitoring. On Days 1, 6, 25, 37, and 45, five samples were randomly collected from each composting pile and then combined to create a homogenised sample for each time point. These time points corresponded to the “Initial”, “Mesophilic”, “Thermophilic”, “Cooling”, and “Mature” stages, respectively, based on composting temperature profiles (Liu et al. 2011; Bao et al. 2021). All homogenised samples were subsequently sub‐divided for chemical and microbial analyses. The composting experiment included 12 replicate piles from the same composting site, resulting in a total of 60 homogenised samples. DOM was sequentially extracted using petroleum ether, n‐hexane, trichloromethane, and methanol, purified with silica gel, and characterised by ESI FT‐ICR MS. Chemical properties, including OM, total nitrogen (TN), total phosphorus (TP), total potassium (TK), available nitrogen (AN), available phosphorus (AP), available potassium (AK), and pH (Table S1), were determined as described in Bao et al. (2021). Because bulk metagenomics is more effective at capturing both lytic and lysogenic phages in environmental samples, particularly lysogenic phages which are often missed by viral metagenomics (Paez‐Espino et al. 2019; Nayfach et al. 2021), bulk genomic DNA was extracted using the Fast DNA SPIN Kit for Faeces for bulk metagenomic sequencing (Josefsen et al. 2015; Zhou et al. 2025).

### 2.2 Bulk Metagenomic Sequencing and Data Processing

Bulk genomic DNA was fragmented to an average size of approximately 400 bp using the Covaris M220 system (Gene Company Limited, China) for paired‐end library preparation. Libraries were constructed using the NEXTflex Rapid DNA‐Seq Kit (Bioo Scientific, Austin, TX, USA), where adapters containing full sequencing primer hybridization sites were ligated to the blunt‐ended fragments. Paired‐end sequencing was performed on an Illumina NovaSeq 6000 platform (Illumina Inc., San Diego, CA, USA) using NovaSeq Reagent Kits, following the manufacturer's protocols, at Majorbio Bio‐Pharm Technology Co. Ltd. (Shanghai, China). In total, approximately 10 Gbp of paired‐end Illumina metagenomic sequencing data were obtained for each sample, resulting in ~600 Gbp of data generated in this study.

Raw sequencing data were processed through the Majorbio Cloud Platform (www.majorbio.com) (Ren et al. 2022). In brief, adapter sequences were removed, and low‐quality reads (length < 50 bp, quality score < 20, or containing ambiguous bases) were filtered out using fastp (version 0.20.0; https://github.com/OpenGene/fastp) (Chen et al. 2018). High‐quality reads were then assembled into contigs with MEGAHIT (k‐mer range: 47–97, step size: 10) based on succinct de Bruijn graphs (Li et al. 2015). Contigs longer than 300 bp were retained for further analysis. Open reading frames (ORFs) were predicted from assembled contigs using MetaGene (Noguchi et al. 2006). ORFs of at least 100 bp were extracted and translated into amino acid sequences according to the NCBI translation table. A non‐redundant gene catalogue was generated using CD‐HIT (version 4.6.1) with a 90% sequence identity and 90% coverage threshold. High‐quality reads were then mapped to the non‐redundant gene catalogue using SOAPaligner (version 2.21) with a minimum 95% identity to calculate gene abundance (Li et al. 2008). Representative sequences from the non‐redundant catalogue were annotated taxonomically by aligning against the NCBI NR database using DIAMOND (version 0.8.35) with an e‐value threshold of 1e^−5^ (Buchfink et al. 2015). Functional annotations were performed by aligning sequences to the eggNOG database for COG classification and to the Kyoto Encyclopedia of Genes and Genomes (KEGG, version 94.2) database, both using DIAMOND with an e‐value cutoff of 1e^−5^. All the bulk metagenomic sequencing data were normalised using the Reads Per Kilobase of transcript per Million mapped reads (RPKM) method (Mortazavi et al. 2008).

### 2.3 Phage Lifestyle Identification

The phage lifestyle of this study was annotated according to Wu, Fang, et al. (2021). Briefly, this was done based on the phage lifestyle dataset construction strategy described by Song (2020), using RefSeq phage genomes from the NCBI database, and labelled according to the bioinformatics method outlined by Mavrich and Hatfull (2017).

### 2.4 Microbial Y‐A‐S Life‐History Strategies

The microbial Y‐A‐S life‐history strategies were classified according to the framework proposed by Malik et al. (2020) and were represented by genome‐level KEGG Orthology (KOs) associated with microbial potential for growth, lignocellulose degradation, and stress tolerance, respectively, based on the KEGG database of whole site community metagenomes (Zheng et al. 2018; Malik et al. 2020; Li et al. 2022). The annotated KOs were presented in Table S2.

### 2.5 Analysis of Microbial‐/Plant‐Derived DOM Components by ESI FT‐ICR MS

The ESI FT‐ICR MS analysis method was described in a previous study (Bao et al. 2021). In brief, the DOM samples were diluted with methanol and analysed using a Bruker Apex 9.4 T Ultra FT‐ICR MS (Bruker, USA), with mass range set at m/z 150–800 and operating parameters following Ikeya et al. (2015), and the resulting molecular formulas were visualised via van Krevelen diagrams and classified into biomolecular groups (i.e., lipid‐like, protein/amino sugar‐like, carbohydrate‐like, unsaturated hydrocarbon‐like, lignin‐like, tannin‐like, and condensed aromatic‐like) based on their O/C and H/C ratios (Kim et al. 2003). Aliphatic molecules (classified as having < 0.3 double bond equivalents (DBEs) per carbon atom and H/C ratio ≥ 1) and terrestrial organic material, such as lignins and tannins, were classified as microbial‐ and plant‐derived molecules respectively, following the approaches of Antony et al. (2014), Feng et al. (2016), and Stubbins et al. (2010).

### 2.6 Statistical Analysis

The random forest (RF) machine learning model, implemented using the rfPermute package (version 1.5.2) in R (Breiman 2001; Archer 2016), was used to identify potential factors predicting the variance in microbial Y/A strategy and microbial‐/plant‐derived DOM component abundance. Spearman correlation analysis (De Winter et al. 2016) was performed to assess the relationships between the ratio of temperate to virulent phage relative abundance and abiotic variables, such as temperature and the chemical properties of the composting samples. Linear regression analysis (Su et al. 2012) was used to evaluate the relationships among temperate/virulent phage relative abundance, microbial Y/A strategy, and microbial‐/plant‐derived DOM components.

## 3 Results and Discussion

### 3.1 Molecular Composition and Characteristics of DOM During Organic Waste Composting

**FIGURE 1:**
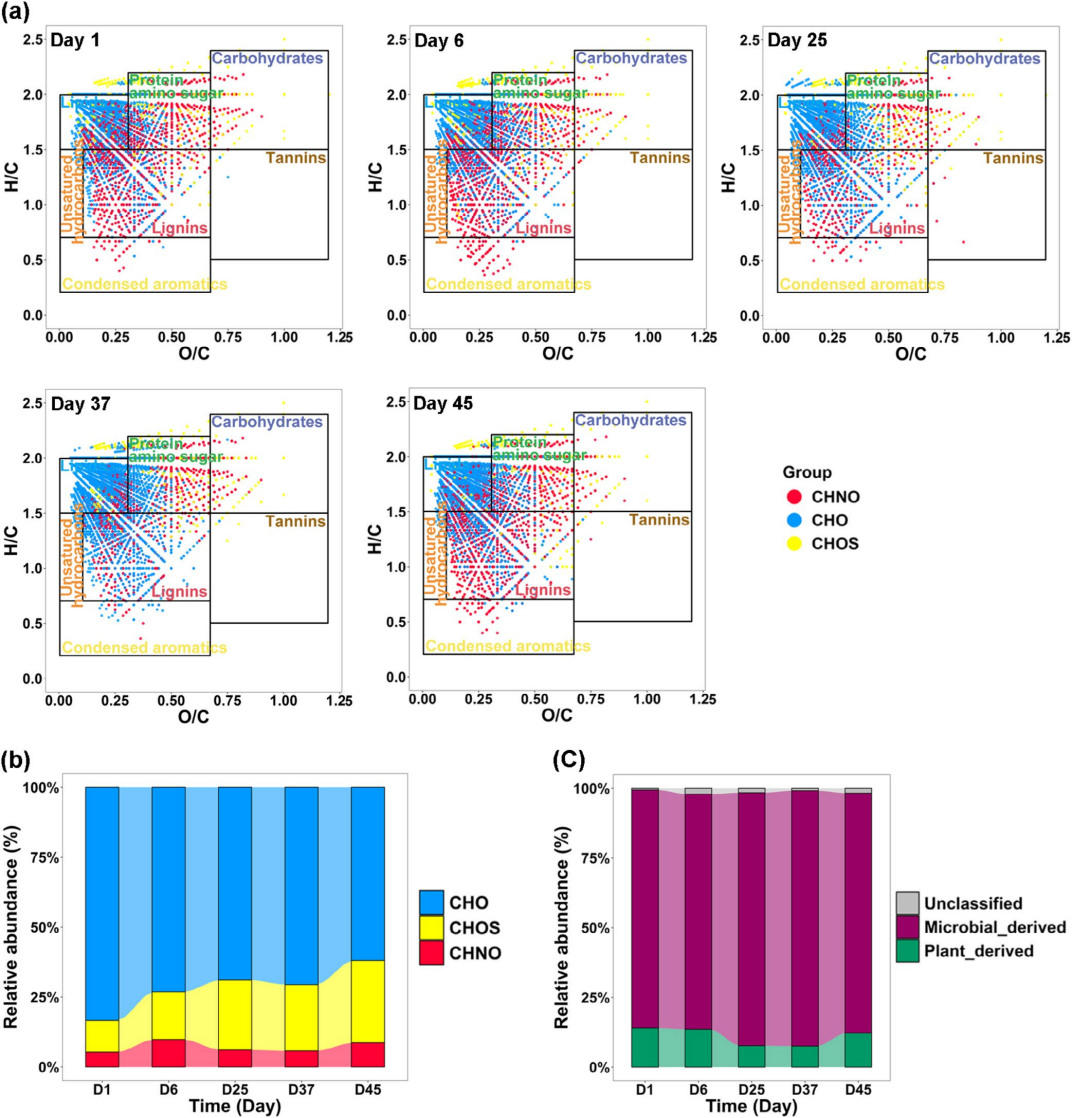
Van Krevelen diagrams of mass spectra from organic waste composting samples (a), bar charts of the major subcategories (b), and the microbial‐ and plant‐derived components of DOM during composting (c) at different composting stages.

ESI FT‐ICR MS was used to analyse the molecular characteristics of DOM extracted from organic waste composting samples (Figure 1). The molecular composition of DOM at each composting stage was visualised using van Krevelen diagrams based on the H/C and O/C ratios (Figure 1a) (Kim et al. 2003). CHO, CHOS, and CHNO were the major subcategories of DOM in the extracts, with average relative abundance of 71.6%, 21.3%, and 7.1%, respectively (Figure 1a,b). The dominance of CHO, similar to that found in cryoconite, suggests that CHO is the primary component of DOM in organic waste (Feng et al. 2016). We further classified the molecular composition of DOM into microbial‐ and plant‐derived components (Feng et al. 2016) and found that the microbial‐derived components were dominant, with relative abundance ranging from 84.3% to 91.6% across the composting periods (Figure 1c).

### 3.2 Trade‐Offs Between Temperate and Virulent Phages, Microbial Y‐ and A‐Strategies, and Microbial‐ Versus Plant‐Derived Components of DOM During Composting

The viral community of the composting samples was dominated by the phylum *Uroviricota*, which represented 87.9% to 98.5% of the total viral community in terms of relative abundance (Figure S1a). The prevalence of *Uroviricota* has also been observed in various other ecosystems, including sheep manure composting (Li et al. 2024), aerobic activated sludge reactors (Tang et al. 2024), and deep‐sea seamount sediments (Yu et al. 2024). At the family level, the viral community of the composting samples was initially diverse, with multiple families including *Herelleviridae* (accounting for an average relative abundance of 1.9% across the entire composting stage), *Drexlerviridae* (1.2%), *Autographiviridae* (1.0%), *Rountreeviridae* (0.9%), *Inoviridae* (0.5%), *Straboviridae* (0.5%), *Steigviridae* (0.4%), *Chaseviridae* (0.3%), *Schitoviridae* (0.2%), and *Suoliviridae* (0.1%) (Figure S1b). *Unclassified Caudoviricetes* (83.3%) and *Unclassified Crassvirales* (2.0%) consistently dominated across all time points. The high proportion of unclassified viruses at the family level is consistent with findings from many viral studies (Jansson and Wu 2023; Yu et al. 2024), and may be partly due to the incompleteness of current taxonomic annotation databases (Jansson and Wu 2023). The top 11 classified viral families were all identified as phages, confirming their dominance within the viral community and highlighting their potential ecophysiological impact on organic waste composting (Zhou et al. 2025).

**FIGURE 2:**
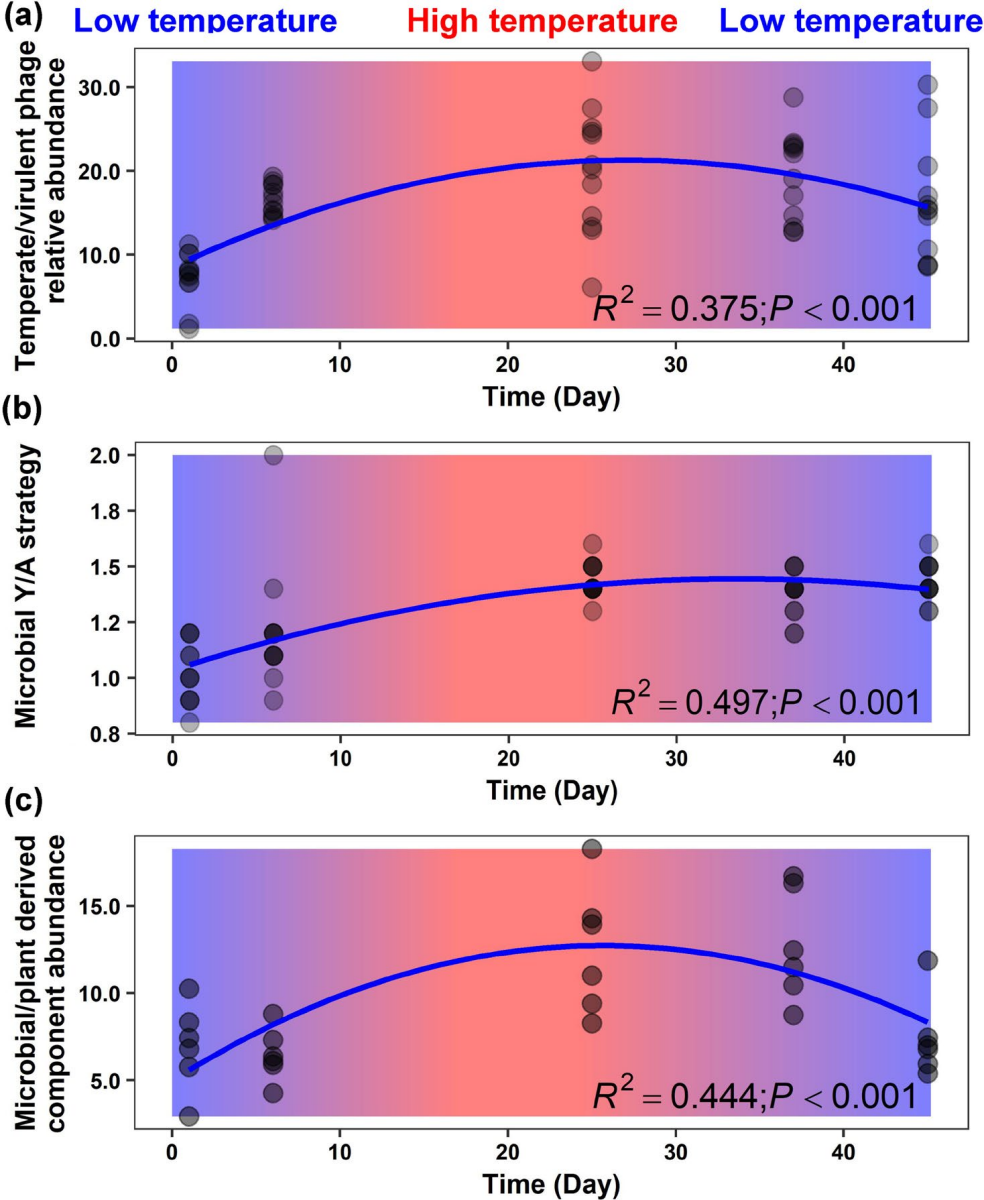
Changing patterns of temperate/virulent phage relative abundance (a), microbial Y/A strategy (b), and microbial‐/plant‐derived DOM components during organic waste composting (c).

To further elucidate phage ecological functions, the phages were classified as temperate or virulent based on their lifestyle at the species level (Figure S1c). Temperate phages dominated throughout the composting period, with their relative abundance decreasing from approximately 70% on Days 1 and 25 to around 20% on Days 37 and 45. In contrast, virulent phages remained at consistently low but detectable levels (average relative abundance 4.8%), with a slight peak on Day 1 (13.1%) followed by a decline thereafter. The dynamic patterns of temperate and virulent phages have not previously been characterised in composting systems. Temperate phages have been reported as the dominant component of viral communities in many natural ecosystems, probably due to their role in enhancing host survival by integrating AMGs into host genomes through lysogenic cycles (Ghosh et al. 2008; Howard‐Varona et al. 2017; Roux and Emerson 2022; Zhao et al. 2024). The ratio of temperate to virulent phages provides a tool to evaluate their potential relative ecological roles in the organic waste composting process (Figure 2a). This ratio increased as thermophilic composting progressed, peaking around Day 25 when the temperature was high and then gradually declined until Day 45 as the temperature decreased (quadratic regression, *R* ^2^ = 0.375, *p* = 0; Figure 2a). Temperature (Spearman correlation coefficient, *r* = 0.40, *p* < 0.01) and resource‐related factors such as OM (*r* = −0.40, *p* < 0.01) and AP (*r* = −0.41, *p* < 0.01) were identified as potential key abiotic environmental drivers influencing the ratio of temperate to virulent phages (Figure S2). These results align with previous observations that environmental factors—such as high resource availability, which favours lytic phages under the “kill‐the‐winner” mechanism (Howard‐Varona et al. 2017; Ji et al. 2023), as well as dry conditions or elevated temperatures can influence the relative dominance and ecological roles of temperate and virulent phages in natural environments (Wu, Davison, et al. 2021; Zhong et al. 2021; Greenrod et al. 2024). These results indicate that composting is associated with a restructuring of the viral community and a shift in the balance between temperate and virulent phages.

To investigate microbial Y‐A‐S life‐history strategies, we selected key KOs associated with these traits (Table S2; Figure S3), which were identified using bulk metagenomic sequencing. The community‐level Y‐strategy increased from Day 1 to 45 (18.9% to 49.1%) (Figure S3). In contrast, the A‐strategy, which initially dominated on Days 1 and 6 (average 43.9%), declined from Day 25 to 45, dropping from 40.2% to 18.6%. The S‐strategy exhibited a gradual decrease throughout the composting process (from 40.2% to 18.6%). A similar trend was observed between the ratio of microbial Y‐ to A‐life‐history strategies and the ratio of temperate to virulent phages relative abundance during organic waste composting, characterised by an initial increase, a peak on Day 25, and a gradual decline by Day 45 (quadratic regression, *R* ^*2*^ = 0.497, *p* = 0; Figure 2b). This pattern suggests a greater importance of the Y‐strategy or reduced importance of the A‐strategy during the mid‐composting stage with higher temperatures, whereas the A‐strategy was more prominent during the initial and final stages. Since the microbial Y‐strategy is closely associated with microbial necromass (Shao et al. 2021), our findings are highly consistent with those of Jia et al. (2024), who also reported that microbial necromass carbon content was low during the initial and final stages, but peaked during the mid‐composting stage of chicken manure composting. Together, these results suggest that composting dynamics shape microbial life‐history strategies, with elevated temperatures favouring the Y‐strategy and promoting necromass accumulation, which may ultimately influence the composition of compost‐derived OM.

The ratio of microbial‐ to plant‐derived DOM components was calculated to reveal the relative importance of each in the composition of the compost product during organic waste composting (Figure 2c). A similar trend was observed as with the ratio of temperate/virulent phage and microbial Y/A strategy (Figure 2c), suggesting that during the mid‐composting stage of composting, the relative contribution of microbial‐derived components to the compost product increases, or the relative contribution of plant‐derived components decreases. In contrast, at the beginning and final stages of composting, the situation is reversed. These findings were also in accordance with the changing patterns of bacterial and fungal necromass carbon among the total organic carbon during chicken manure composting (Jia et al. 2024).

### 3.3 Linkages Among Phage Types, Microbial Y‐A‐S Life‐History, and DOM Components During Composting

**FIGURE 3:**
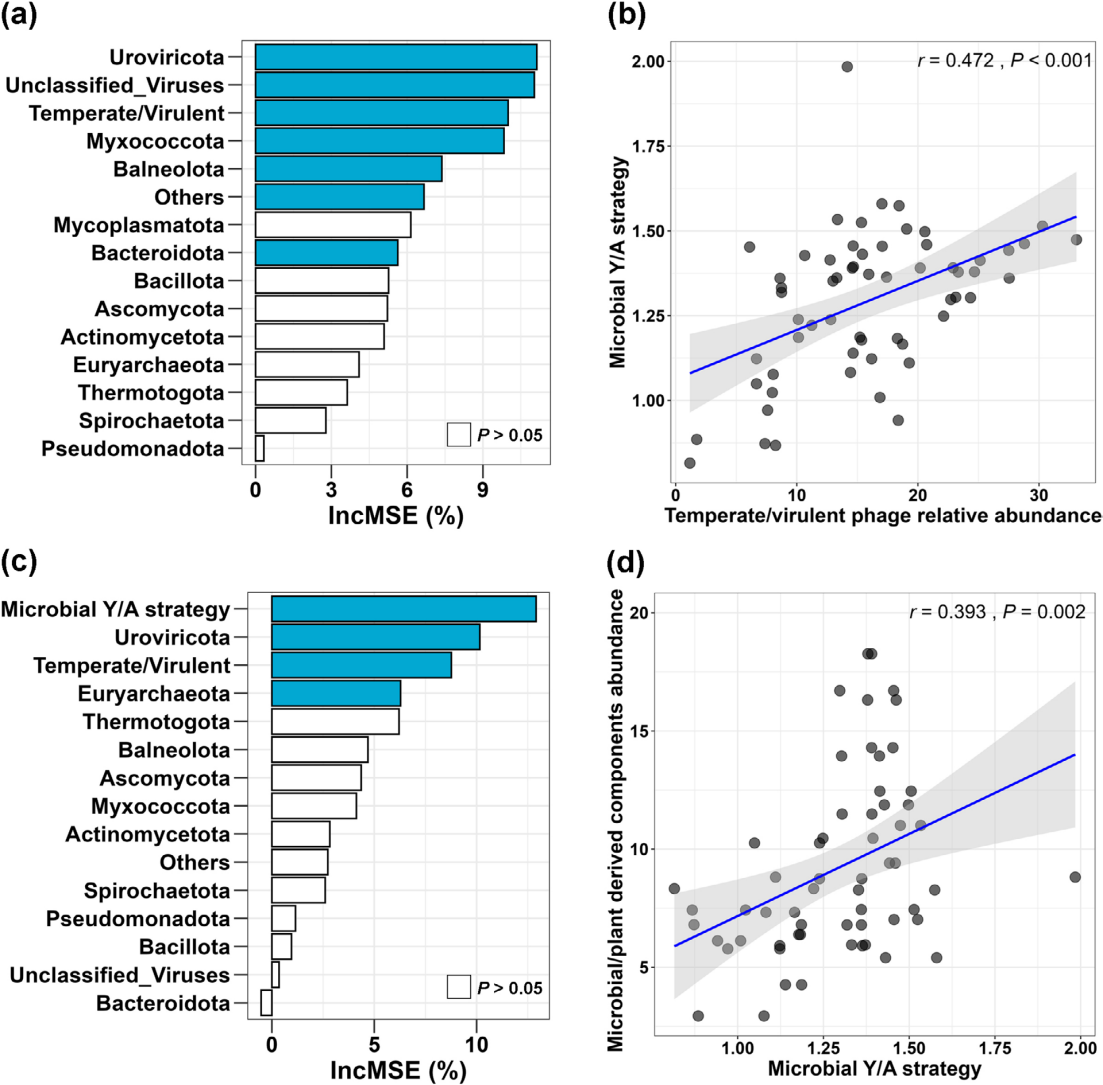
Potential drivers of variation in microbial Y/A strategy (a) and microbial‐/plant‐derived DOM components (c) revealed by RF machine learning model, and linear regression analyses linking temperate/virulent phage relative abundance, microbial Y/A strategy, and microbial‐/plant‐derived DOM components (b, d).

We used the RF machine learning model to identify potential factors for predicting the variance in microbial Y/A strategy (Figure 3a) and microbial‐/plant‐derived DOM components abundance (Figure 3c) during organic waste composting. Viruses, such as *Uroviricota* and *Unclassified Viruses*, were found to be important biomarkers for predicting microbial Y/A strategy variance, accounting for 11.1% and 11.0%, respectively (*p* < 0.05; Figure 3a). Since viral classification at the phylum level does not accurately distinguish between temperate and lytic life history types, we also included temperate/virulent phages relative abundance in the RF machine learning model. This inclusion also effectively predicted microbial Y/A strategy variance during organic waste composting, accounting for 10.0% (*p* < 0.05; Figure 3a). These results are consistent with the premise that phages can regulate host growth (associated with the Y‐strategy) or metabolism (associated with the A‐strategy) through their lysogenic and lytic lifestyles, respectively (Quesada et al. 2012; Huang et al. 2021; Albright et al. 2022; Jansson and Wu 2023). Moreover, the microbial Y/A strategy was identified as the most important factor in predicting the variance in the abundance of microbial‐/plant‐derived DOM components during organic waste composting, explaining 12.9% of the variation (*p* < 0.05; Figure 3c). Linear regression analysis revealed significant positive associations between the relative abundance of temperate/virulent phages, microbial Y/A strategy, and the abundance of microbial‐/plant‐derived DOM components (*r* = 0.393 and 0.472, *p* < 0.05) (Figure 3b,d; Figure S4a), as well as a significant positive association between the relative abundance of temperate/virulent phages and OM contents (*r* = −0.421, *p* < 0.001) (Figure S4b).

**FIGURE 4:**
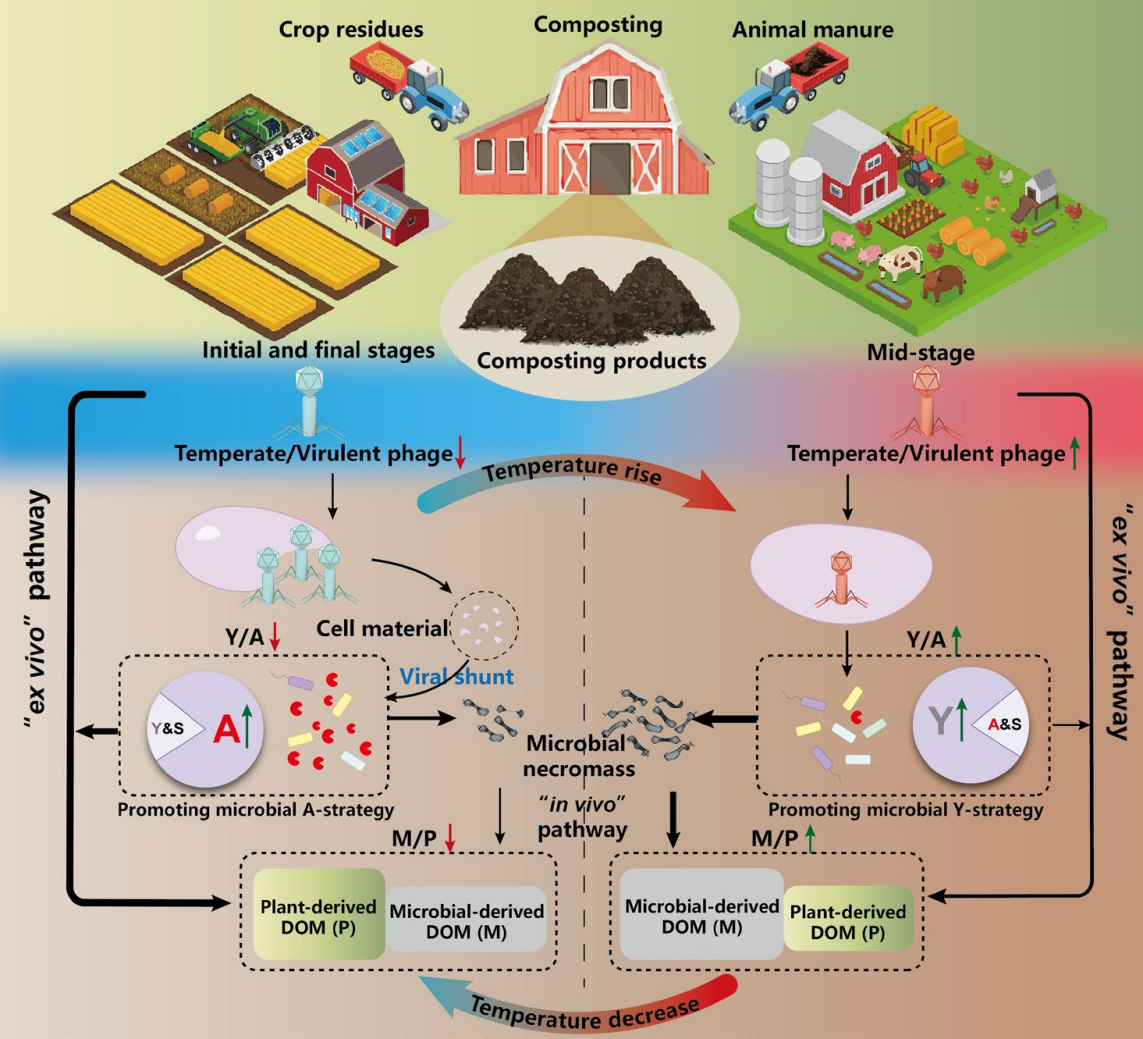
Mechanistic framework of temperate‐ and virulent phage‐driven trade‐offs among microbial Y‐A‐S life‐history strategies shaping DOM component transformation during organic waste composting.

Collectively, these results are in agreement with the hypothesis that increased relative importance of temperate phages, such as during the mid‐composting stage when temperatures are high, promotes the microbial Y‐strategy, resulting in the accumulation of microbial‐derived DOM components in the compost product through the in vivo pathway (Liang et al. 2017; Malik et al. 2020; Shao et al. 2021; Zheng et al. 2021; Jansson and Wu 2023) (Figure 4). Conversely, the prevalence of virulent phages during the initial and final stages with lower temperatures promotes the microbial A‐strategy, resulting in a relatively greater accumulation of plant‐derived DOM components in the compost product via ex vivo pathway (Figure 4). These findings reveal how phage‐mediated shifts in microbial life‐history strategies shape DOM composition during composting and may provide guidance for practical process optimization. Maintaining thermophilic conditions that favour temperate phages may enhance the accumulation of more stable, microbially derived DOM and thereby improve compost quality, whereas conditions that favour virulent phages may increase the proportion of more labile, plant‐derived components. Thus, the ratios of temperate to virulent phages, microbial Y‐ to A‐strategies, and microbial‐ to plant‐derived DOM may serve as useful early indicators for monitoring and improving compost production.

Our finding supports the first hypothesis that virulent phages suppress the Y‐strategy and reduce microbial‐derived components to compost, but do not support the second hypothesis, which posits that temperate phages promote the A‐strategy and enhance plant‐derived components. According to the “kill‐the‐winner” model associated with virulent phages (Winter et al. 2010), hosts can be lysed due to viral infection, releasing cellular material into the environment. This material can then be metabolised by other environmental microbial populations via the “viral shunt” pathway, thereby increasing their metabolic activity (Pratama and van Elsas 2018; Jansson and Wu 2023). In accordance with this paradigm, increased virulent phage lysis and environmental microbial metabolic activity have been observed, for example, during permafrost thaw and with differences in historical precipitation (Emerson et al. 2018; Wu, Davison, et al. 2021). Correspondingly, we observed that the increased relative importance of virulent phages during the initial and final stages could promote the relative importance of microbial A‐strategy and plant‐derived DOM components in the compost product (Figures 1, 2, 3), supporting the first hypothesis.

For the second hypothesis, the “piggyback‐the‐winner” model associated with temperate phages seems too narrow. There is no reason why only metabolism‐related AMGs, such as resource acquisition abilities (linked to the A‐strategy) should be integrated into the host genome. Also microbial growth‐related AMGs can be integrated and expressed in host genomes through lysogeny (Zheng et al. 2022). This could explain the increase in the relative importance of temperate phages during the mid‐composting stage did not enhance the microbial A‐strategy, but rather the Y‐strategy, leading to the increased relative importance of microbial‐derived DOM components in the compost product (Figures 1, 2, 3).

## 4 Conclusions

The findings presented here suggest that shifts in the relative importance of temperate and virulent phages drive trade‐offs in microbial life‐history strategies, thereby influencing component transformation during organic waste composting. A rising prevalence of temperate phages promotes the microbial Y‐strategy, facilitating the accumulation of microbial‐derived DOM components in the compost product, whereas an increasing dominance of virulent phages favours the microbial A‐strategy, leading to the enrichment of plant‐derived DOM components. Given that microbial‐derived components are more stable in soil and contribute to the maintenance of soil organic carbon, while plant‐derived components are more readily degradable and available for crop uptake (Liang et al. 2017; Zheng et al. 2021), our study provides new ecological insights into how phage‐mediated microbial life‐history strategies shape compost products and, consequently, their functional roles in agricultural soil ecosystems. To provide more direct evidence beyond the correlation analyses employed here, future research should incorporate controlled experiments during organic waste composting, such as DNA stable‐isotope probing‐based metagenomic sequencing, enzymatic activity assays, and targeted inoculations with active or inactive temperate and virulent phages.

## Author Contributions

Yuanyuan Bao: conceptualization, methodology, software, data curation, investigation, validation, formal analysis, visualisation, funding acquisition, writing – original draft, writing – review and editing. Jan Dolfing: investigation, formal analysis, supervision, writing – review and editing. Ruirui Chen: investigation, formal analysis, methodology. Chongwen Qiu: methodology, data curation, formal analysis, investigation, visualisation, project administration, resources. Jianwei Zhang: software, formal analysis, data curation, methodology, investigation. Xin Zhou: formal analysis, investigation, software. Liang Liu: software, formal analysis, investigation. Yiming Wang: methodology, formal analysis, investigation, validation, project administration, resources. Xiangui Lin: methodology, validation, investigation, supervision, resources, project administration, formal analysis. Youzhi Feng: investigation, supervision, resources, project administration, funding acquisition.

## Funding

This work was supported by the National Natural Science Foundation of China (Project No. 42577330, 42207365, 42577352, and 42177297), the Natural Science Foundation of Jiangsu Province (Project No. BK20221161), the Chinese Academy of Sciences (CAS) Strategic Priority Research Program (grant no. XDA28010302).

## Conflicts of Interest

The authors declare no conflicts of interest.

## Supporting information


**Table S1:** The chemical and physical properties of samples during composting (Bao et al. 2021).
**Figure S1:** Viral community composition during organic waste composting at the phylum (a) and family (b) levels, and phage lifestyle composition at the species level (c). “Others” and “Unclassified_Phage_lifestyle” represent low‐abundance classifications (< 0.077% in a and < 0.068% in b) and viruses lacking temperate or virulent lifestyle annotations, respectively.
**Figure S2:** Spearman correlations between the ratio of temperate to virulent phage relative abundance and abiotic variables, such as temperature and the chemical properties of the composting samples, are shown. The numbers in the plot represent the Spearman correlation coefficients, with red and blue indicating positive and negative correlations, respectively. “*”, “**”, and “***” denote significance levels at *p* < 0.05, *p* < 0.01, and *p* < 0.001, respectively.
**Figure S3:** Shifts in microbial life‐history strategies during organic waste composting.
**Figure S4:** Relationships between temperate/virulent phage relative abundance and microbial−/plant‐derived DOM components (a) and OM contents (b).

**Table S2:** KOs related to microbial Y, A, and S life‐history strategy.

## Data Availability

The shotgun metagenome datasets of this study were deposited into the DNA Data Bank of Japan (DDBJ) database (https://www.ddbj.nig.ac.jp/index‐e.html) under accession number DRA020578. All other data generated or analysed during this study are included in this published article or are available from the corresponding author upon reasonable request.
