# Supplementary material for: Phages Shape the Transformation of Organic Matter During Composting
Source: Microb Biotechnol. 2025 Dec 21;18(12):e70291. doi: 10.1111/1751-7915.70291 (PMC12719608; doi:10.1111/1751-7915.70291)
Supplement: Supplementary file 1 — Table S1: The chemical and physical properties of samples during composting (Bao et al. 2021). Figure S1: Viral community composition during organic waste composting at the phylum (a) and family (b) levels, and phage lifestyle composition at the species level (c). “Others” and “Unclassified_Phage_lifestyle” represent low‐abundance classifications (< 0.077% in a and < 0.068% in b) and viruses lacking temperate or virulent lifestyle annotations, respectively. Figure S2: Spearman correlations between the ratio of temperate to virulent phage relative abundance and abiotic variables, such as temperature and the chemical properties of the composting samples, are shown. The numbers in the plot represent the Spearman correlation coefficients, with red and blue indicating positive and negative correlations, respectively. “*”, “**”, and “***” denote significance levels at p < 0.05, p < 0.01, and p < 0.001, respectively. Figure S3: Shifts in microbial life‐history strategies during organic waste composting. Figure S4: Relationships between temperate/virulent phage relative abundance and microbial−/plant‐derived DOM components (a) and OM contents (b). [file MBT2-18-e70291-s002.docx]

**Supplementary material 1:**

**Table S1.** The chemical and physical properties of samples during composting ([Bao et al., 2021](#_ENREF_4)).

| Time (Day) | OM (%) | TN (%) | TP (%) | TK (%) | AN (g/kg) | AP (g/kg) | AK (g/kg) | pH | C/N | Temperature (°C) |
| --- | --- | --- | --- | --- | --- | --- | --- | --- | --- | --- |
| 1 | 69.3±1.3c | 1.6±0.2a | 0.7±0.1a | 3.3±0.5a | 4.5±1.6b | 3.8±0.2b | 27.4±3.7a | 8.4±0.4a | 24.9±4.2ab | 32 |
| 6 | 66.5±1.7b | 1.3±0.2b | 0.7±0.2a | 3.6±0.4ab | 4.5±1.8b | 3.3±0.5a | 29.8±3ab | 8.8±0.6ab | 30.1±4.2b | 63 |
| 25 | 63.7±2.3a | 1.4±0.3b | 0.8±0.1a | 3.5±0.7a | 2.9±0.6a | 3.3±0.3a | 30.2±2.3ab | 8.9±0.3b | 26.8±5.9ab | 76 |
| 37 | 62.3±1.5a | 1.7±0.3b | 0.8±0.2a | 4±0.5b | 2.5±0.5a | 3.6±0.2ab | 31.8±2.4b | 8.4±0.3a | 21.5±3.9a | 74 |
| 45 | 61.2±1.1a | 1.6±0.3b | 0.8±0.2a | 3.8±0.7ab | 3.1±0.8ab | 3.7±0.4b | 31.1±2.3ab | 8.9±0.3ab | 22.9±5.3a | 30 |

Different letters denote significant differences (*P* < 0.05).

**
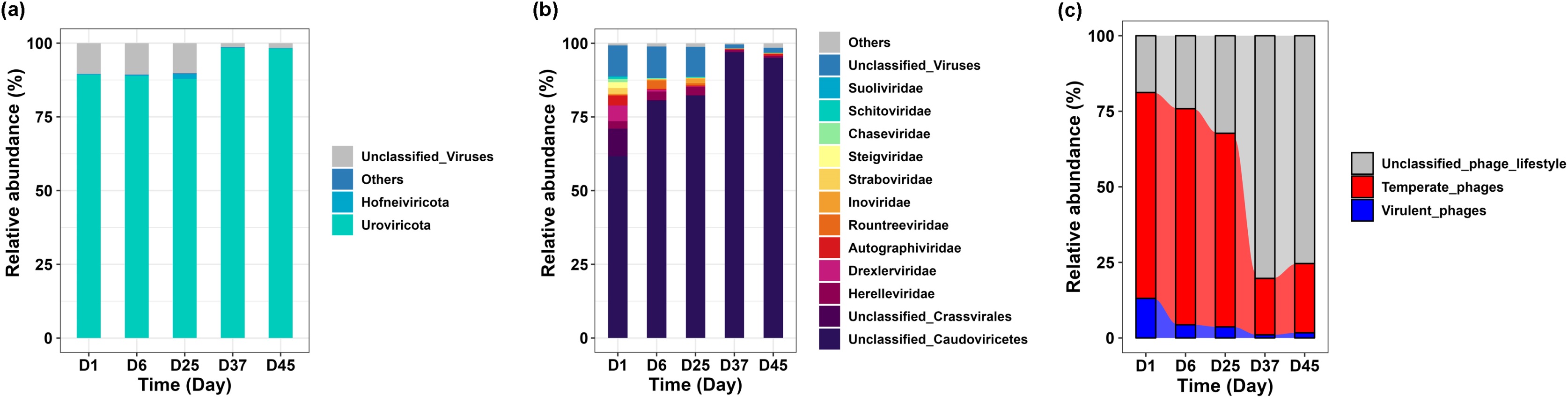
**

**Figure S1.** Viral community composition during organic waste composting at the phylum (a) and family (b) levels, and phage lifestyle composition at the species level (c). “Others” and “Unclassified_Phage_lifestyle” represent low-abundance classifications (<0.077% in a and <0.068% in b) and viruses lacking temperate or virulent lifestyle annotations, respectively


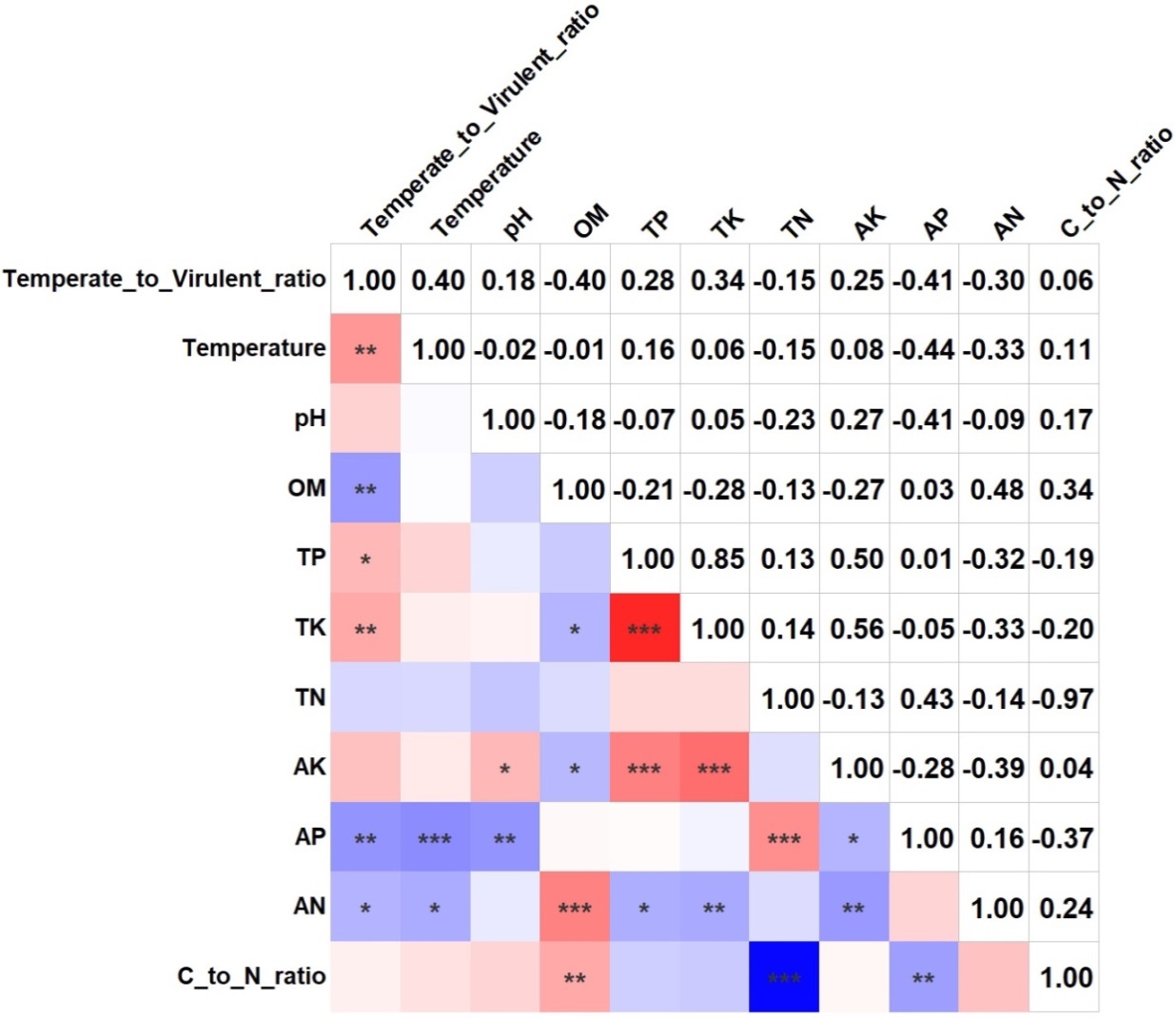


**Figure S2.** Spearman correlations between the ratio of temperate to virulent phage relative abundance and abiotic variables, such as temperature and the chemical properties of the composting samples, are shown. The numbers in the plot represent the Spearman correlation coefficients, with red and blue indicating positive and negative correlations, respectively. “*”, “**”, and “***” denote significance levels at *P* < 0.05, *P* < 0.01, and *P* < 0.001, respectively.


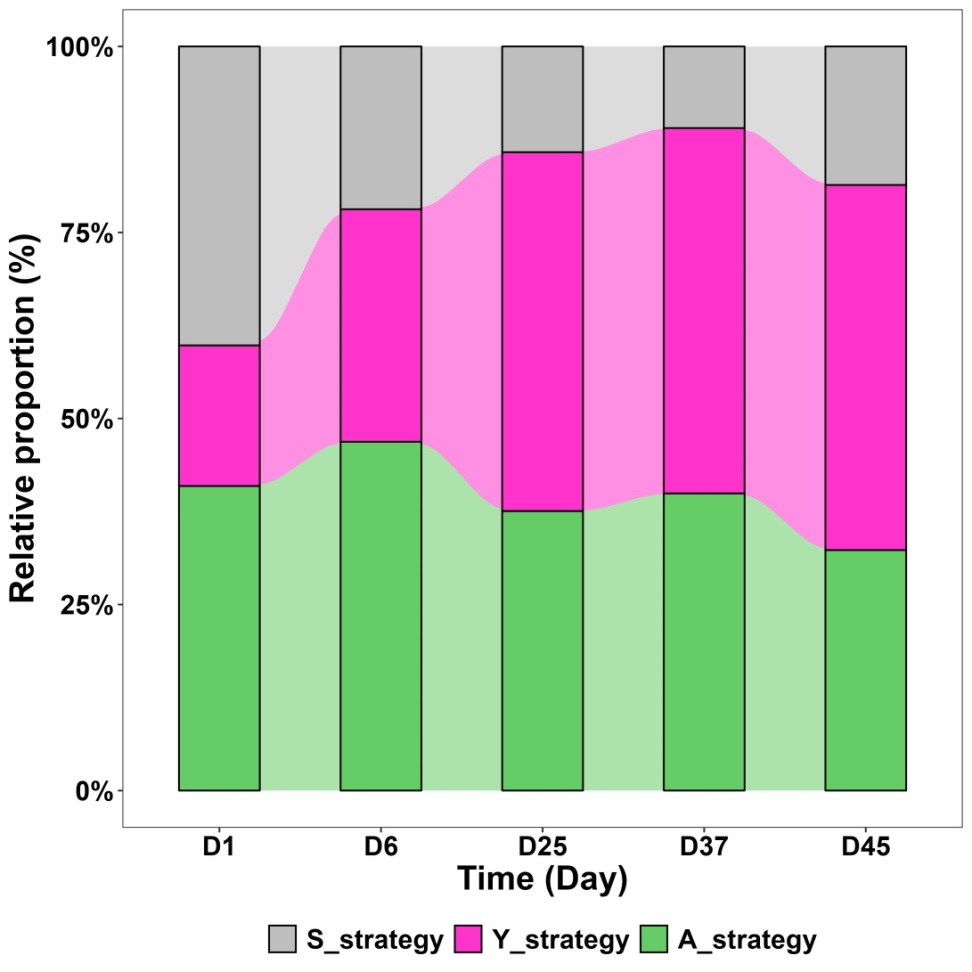


**Figure S3.** Shifts in microbial life-history strategies during organic waste composting.


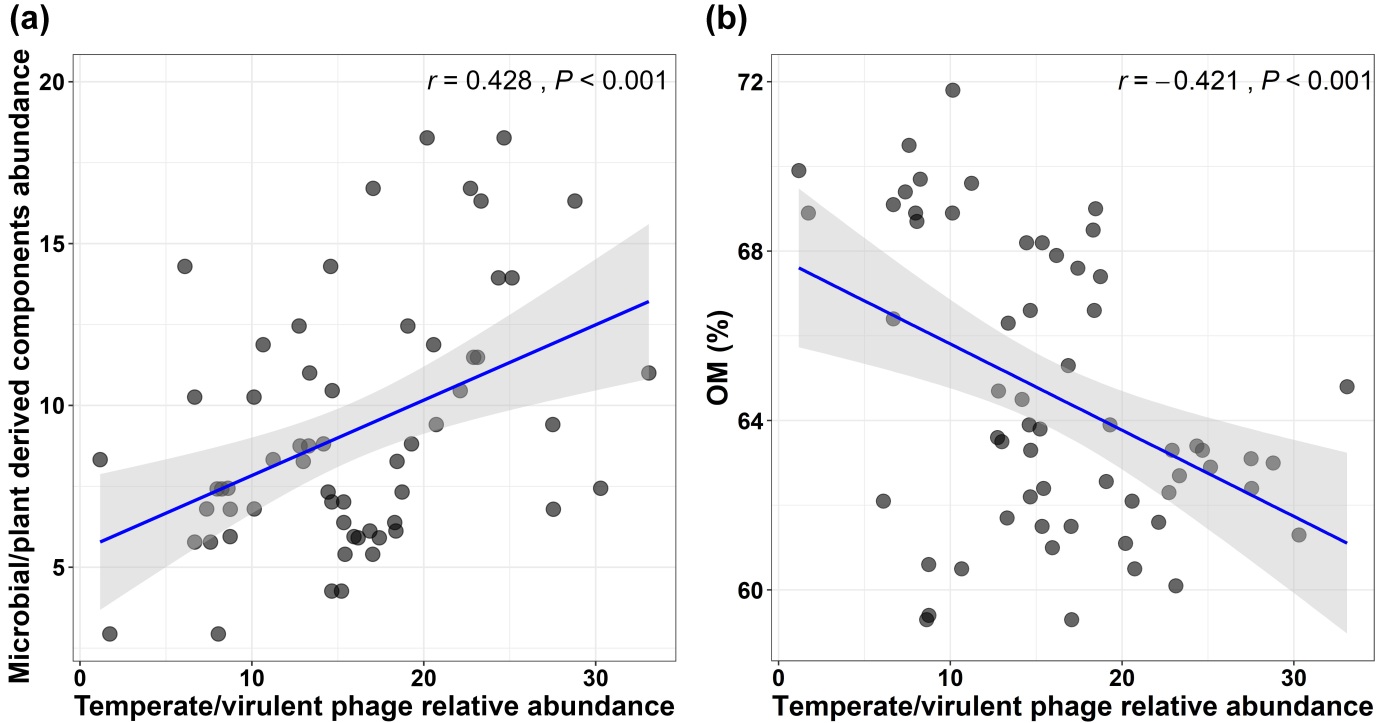


**Figure S4.** Relationships between temperate/virulent phage relative abundance and microbial-/plant-derived DOM components (a) and OM contents (b).

**Reference**

Bao, Y.Y., Feng, Y.Z., Qiu, C.W., Zhang, J.W., Wang, Y.M., Lin, X.G., 2021. Organic matter- and temperature-driven deterministic assembly processes govern bacterial community composition and functionality during manure composting. Waste Manage. 131, 31-40.
